# Decreasing Resistivity of Silicon Carbide Ceramics by Incorporation of Graphene

**DOI:** 10.3390/ma13163586

**Published:** 2020-08-13

**Authors:** Ningning Cai, Daidong Guo, Guoping Wu, Fangmin Xie, Shouhong Tan, Nan Jiang, He Li

**Affiliations:** 1 Key Laboratory of Marine New Materials and Related Technology, Zhejiang Key Laboratory of Marine Materials and Protection Technology, Ningbo Institute of Material Technology & Engineering, Chinese Academy of Sciences, Ningbo 315201, China; cainingning0311@163.com (N.C.); guodaidong@nimte.ac.cn (D.G.); lihe@nimte.ac.cn (H.L.); 2 Ningbo FLK Technology CO. LTD, Ningbo 315104, China; wgp@sealmann.com (G.W.); xfm@sealmann.com (F.X.); 3 Shanghai Institute of Ceramics, Chinese Academy of Sciences, Shanghai 200050, China

**Keywords:** SiC ceramics, graphene, volume resistivity, bulk density, flexural strength

## Abstract

Silicon carbide (SiC) ceramic is an ideal material for mechanical seal because of its super hardness, high strength, low friction coefficient, good thermal conductivity, and resistance to friction and wear. However, due to relatively high resistivity of SiC ceramic, the triboelectric charge caused by rubbing of mechanical seal end-faces could not be released. It is terrible that the accumulation of triboelectric charge could cause electrochemical corrosion, which would accelerate wear. To decrease the resistivity of SiC ceramic is a desire for improving the performance of mechanical seal. In this research, decreasing resistivity of pressureless sintered SiC ceramic was investigated by conductive pathways through semiconductive grains in a body by incorporation of graphene, which has an extremely low resistivity. With the increasing of graphene from 0 to 2 wt.%, the volume resistivity of SiC ceramics sintered with graphene decreased logarithmically from >10^6^ to around 200 Ω·cm, and the bulk density decreased gradually, from 3.132 to 3.039 g/cm^3^. In order to meet the requirements of mechanical seal, SiC ceramic sintered with 1 wt.% of graphene, for which the volume resistivity is of 397 Ω·cm, the bulk density is of 3.076 g/cm^3^, and the flexural strength is of 364 MPa, was optimized when all properties were taken into consideration. It is possible to fabricate low-resistivity SiC ceramic as a useful friction pair material for mechanical seal in a special condition, without excessive loss of their excellent mechanical properties by the introduction of partially connected graphene as conductive pathway into semiconducting ceramic.

## 1. Introduction

Silicon carbide ceramic (SiC ceramic) is one of the most widely used high-temperature structural ceramics, because of its high mechanical strength, high hardness, high-temperature strength, excellent thermal conductivity, resistance to friction and wear, resistance to oxidation, resistance to corrosion, and low specific gravity. Meanwhile, electrically conductive SiC ceramic is highly desired for some industrial applications, such as semiconductor manufacturing. 

For mechanical seals in a special condition, SiC ceramic, which is a very suitable material for friction pair, is required to have a resistivity less than 1 × 10^3^ Ω·cm to conduct the triboelectric charge, which is generated from the rubbing of end-faces in operation and which could cause electrochemical corrosion. Meanwhile, SiC ceramic as a friction pair material is also required to have a flexural strength of more than 350 MPa and a bulk density more than 3.05 g/cm^3^, in order to provide sufficient mechanical performance. However, the resistivity of conventional SiC ceramics is 1 × 10^3^ to 1 × 10^5^ Ω·cm; thus, decreasing the resistivity of SiC ceramics while retaining the material’s excellent mechanical properties is of great interests these applications.

To improve the electrical conductivity to SiC ceramic, dispersions of conducting second phase particles have been reported. In such composites, conducting particle contents above 12–30 vol% is necessary to produce conducting ceramics. However, the addition of large amounts of a second phase can potentially compromise the excellent mechanical properties of the matrix ceramics. Therefore, people are seeking ways to decrease the electrical resistivity of SiC ceramics by less addition of the second phase [1,2,3]. Another problem in the previous reports was that the ceramics were often sintered by hot-press sintering [4,5,6,7]. Although hot-pressing sintering was a widely used method to promote the densification of SiC ceramic, it requires costly equipment and can only produce SiC ceramics with limited geometry. Therefore, hot-press sintering is not suitable for industrial production of friction pair used for mechanical seals. 

Graphene is considered one of the strongest materials with exceptional charge transport, thermal, optical, and mechanical properties [8], because of its excellent two-dimensional honeycomb lattice structure, which is closely packed with carbon atoms. These properties make it an ideal filler in the fabrication of conducting and robust polymers and ceramic composites [9,10,11,12,13,14,15,16]. The graphene can be introduced into SiC ceramic by sublimating the silicon from silicon carbide in a controlled atmosphere [11,17]. This kind of graphene, usually formed and present on the surface of SiC single or polycrystalline, could seriously regulate the surface resistivity. However, the surface of mechanical seal is often damaged due to friction and wear during application. Therefore, for mechanical seals, volume resistivity is usually considered rather than surface resistivity. Another way to introduce graphene into SiC ceramic is by adding graphene by uniform mixing and sintering. It has been indicated that graphene can improve the electrical conductivity of ceramic composites, such as Al_2_O_3_ [18], SiO_2_ [19], ZrB_2_ [20], and Si_3_N_4_ [21] composites. However, reports on graphene decreasing resistivity of SiC composite are relatively few. Guo et al. [22] investigated the effect of graphene on the microstructure, mechanical, and tribological properties of SiC ceramic, but their mechanisms were not discussed. 

In this study, the pressureless sintering was used to guarantee production efficiency and production cost. To decrease the resistivity of SiC ceramics without losing its excellent mechanical properties for mechanical seals, the amount of graphene was limited and optimized. The effect of graphene as dopants on the flexural strength, bulk density, and volume resistivity, which were crucial properties of SiC ceramic for mechanical seals, was investigated, and their mechanisms were preliminarily explored. 

## 2. Materials and Methods 

Uniform graphene suspension was obtained by emulsification and ultrasound dispersing graphene powder (EPOG-80, Ningbo Morsh Tech Co., Ltd., Ningbo, China) and carboxymethyl cellulose sodium (CMC-Na, N = 500, Sinopharm Chemical Reagent Co., Ltd, Shanghai, China), with the weight ratio of 10:1, into deionized water. 

Commercially available SiC powder (size of 0.5–5 μm) as starting powder, B_4_C powder (size of 0.5–5 μm) as sintering additive with the weight ratio of 100:1, and various amounts of graphene suspension and ethanol were mixed by conventional wet-ball-milling with SiC balls, for 24 h, to obtain a homogeneous mixture. 

The solvent was evaporated from the mixture at 90 °C. The prepared mixture was dried at 120 °C, and then it was dry-ball-milled for 6 h to eliminate hard agglomerations before sieving through a 74 μm mesh. The obtained powder compositions were dry-pressed at 50 MPa and then cold-isostatic-pressed at 200 MPa, to obtain a denser ceramic billet. 

The ceramic billet was dewaxed at 800 °C for 2 h, under around 10 Pa of vacuum atmosphere in the pre-firing furnace, and then pressureless sintered at 2050 °C for 2 h, under the protection of argon gas, in the final firing furnace. 

The content of graphene in the SiC ceramics was adjusted to 0.1, 0.5, 1, and 2 wt.%. The sample was labeled as 0.1 M, 0.5 M, 1 M, and 2 M respectively. SiC ceramic without graphene was also prepared as the control group and labeled as 0 M. 

The bulk density of graphene-doped SiC ceramics was measured by the Archimedes method via immersion in deionized water. The theoretical density was calculated by using the specific gravity of 3.21 for SiC. 

The flexural strength of the samples was determined by Three-Point Bending Test on an Instron Materials Testing Machine (Instron 5566, Instron Co., Ltd, Norwood, MA, USA). At least 5 specimens were tested for each sample, to determine an average of flexural strength. The specimen thickness, width, span length, and crosshead speed were 3 mm, 4 mm, 30 mm, and 0.5 mm/min, respectively. 

The volume resistivity of the samples was measured by 2-probe method, using a Keithley electrometer (model 2001, Keithley Instruments Inc., Cleveland, Ohio, USA) at room temperature. The upper and lower side of samples with size of Φ28 mm × h1.5 mm were polished and painted with silver paste, to improve the electrical contacts between the copper wires and the sample.

The microstructure of the samples was observed by scanning electron microscopy (SEM, Model EVO18, ZEISS, Jena, Germany) on polished fracture surfaces, to reveal the morphologies and locations of SiC grains and grain boundary phases. 

The chemical composition and elements distribution on cross-sections of the samples were determined by Electron Probe Microanalyzer (EPMA-1720, SHIMADZU, Kyoto, Japan). A user-defined image area of 30 μm × 30 μm was employed for the element mappings.

Raman spectra of the samples were recorded by confocal microscope Raman spectrometer (inVia Reflex, RENISHAW, Gloucester, UK), using the 532 nm laser wavelength excitation, and an acquisition up to 1800 cm^−1^. 

## 3. Results and Discussions

### 3.1. Flexural Strength

**Figure 1:**
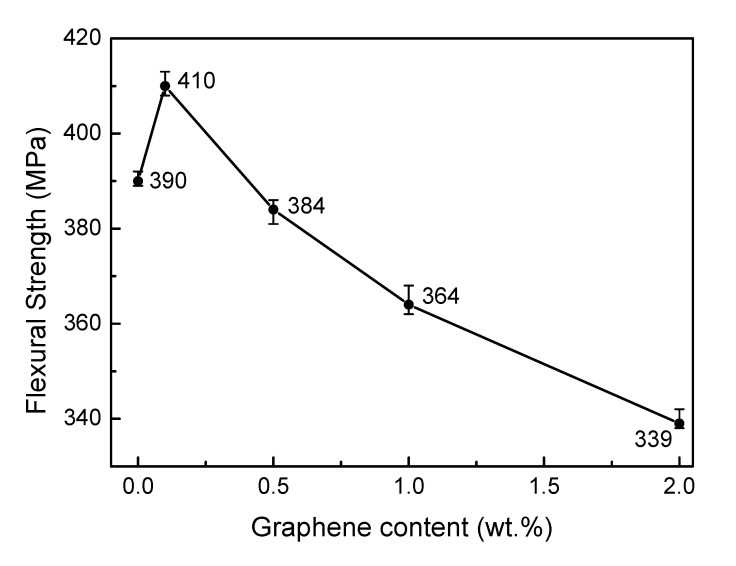
Effect of graphene doping on flexural strength of SiC ceramics.

The flexural strength of SiC ceramics with the influence of the graphene doping amount is shown in Figure 1. With the increasing of graphene amount, the flexural strength of SiC ceramics first increased and then decreased. The flexural strength of 0 M is 390 MPa. The flexural strength of 0.1 M was the highest among all samples, at 410 MPa, which increased 5% of 0 M. Further increase the graphene amount resulted in the gradual decrease of the flexural strength of SiC ceramics. When the graphene amount reached 2 wt.%, the flexural strength of 2 M was less than 90% of 0 M.

### 3.2. Density

**Figure 2:**
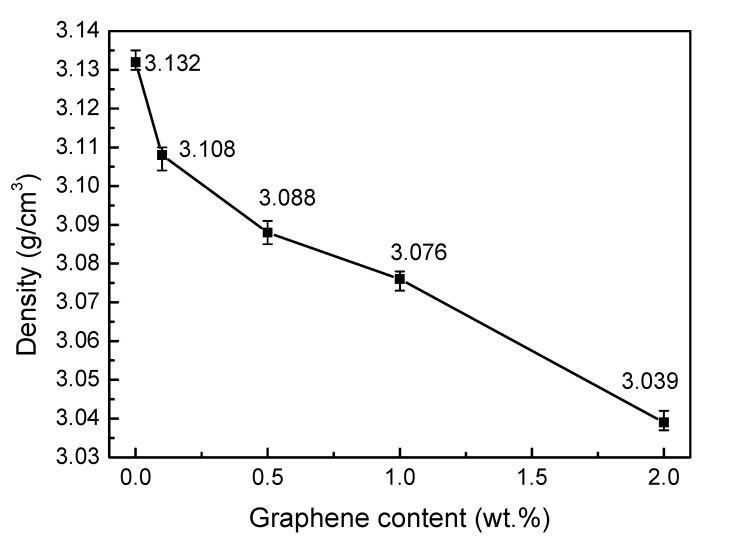
Effect of graphene doping on the density of SiC ceramics.

The influence of the graphene doping amount on the bulk density of SiC ceramics is shown in Figure 2. With the increase of the graphene doping amount, the bulk density of SiC ceramics showed a trend of gradual decrease. Overall, 0 M has the highest bulk density, at 3.132 g/cm^3^. The bulk density of 0.1 M decreased to 99.23% of the original density. When the amount of doped graphene reached 2 wt.%, the bulk density of 2 M was only 3.039 g/cm^3^, which cannot meet the requirement of friction pair materials for mechanical seals. The bulk density of 1 M was 3.076 g/cm^3^, which just slightly exceeded the required density, 3.05 g/cm^3^. Therefore, the graphene should not be more than 1 wt.%.

### 3.3. Volume Resistivity

**Figure 3:**
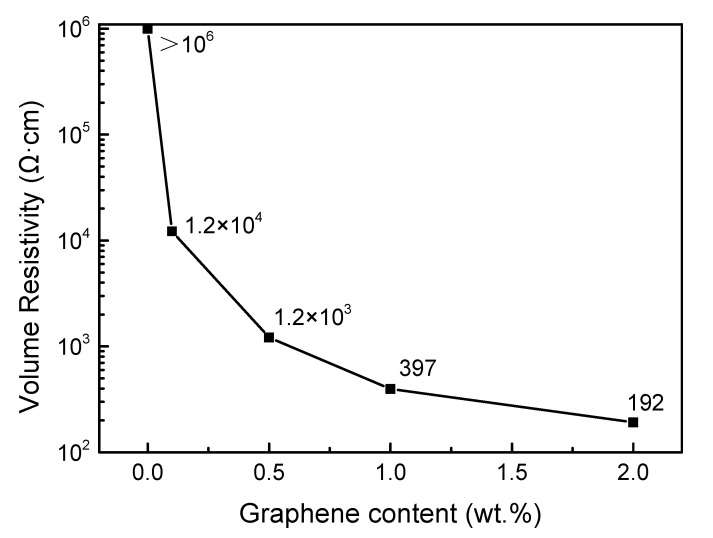
Effect of graphene doping on volume resistivity of SiC ceramics.

The influence of graphene on the volume resistivity of SiC ceramics is shown in Figure 3. With the increase of graphene, the volume resistivity of SiC ceramics decreased logarithmically. At room temperature, 4H-SiC single crystal is a typical wide-band gap semiconductor material with the band gap width of 3.23ev [23]. However, as a polycrystalline material, SiC ceramics contain secondary phases, including grain boundary, pores, impurities, etc. Meanwhile the orientation of grain was not consistent, resulting in a very complex internal microstructure and a large range of volume resistivity fluctuation. The volume resistivity of 0 M was above 10^6^ Ω·cm, which was much larger than the target resistivity, 1 × 10^3^ Ω·cm. When a small amount of graphene was doped into SiC ceramics, the resistivity of 0.1 M was two orders of magnitude lower than that of 0 M. When the graphene reached 1 wt.%, the resistivity of 1 M was 397 Ω·cm, which meets the requirements of the resistivity for mechanical seal. When more graphene was doped, the resistivity decrease was less sensitive to the graphene amount, and the resistivity of 2 M was 192 Ω·cm, half of that of 1 M. 

Table 1 summarizes the bulk density, flexural strength, and volume resistivity of SiC ceramics with various doped amounts of graphene. When the doping amount of graphene was <1 wt.%, as the doped amount of graphene increased, the volume resistivity and density of SiC ceramics decreased. When the doped amount of graphene was >1 wt.%, the volume resistivity of SiC ceramics tended to change gradually, but its density and flexural strength were reduced to below the requirements of friction pair materials for mechanical seals. The 1 M doped with 1 wt.% graphene met not only the resistivity requirements, but also the density and flexural strength requirements. Therefore, 1 M was an ideal candidate as the friction pair materials for mechanical seals. 

### 3.4. Microstructure

**Figure 4:**
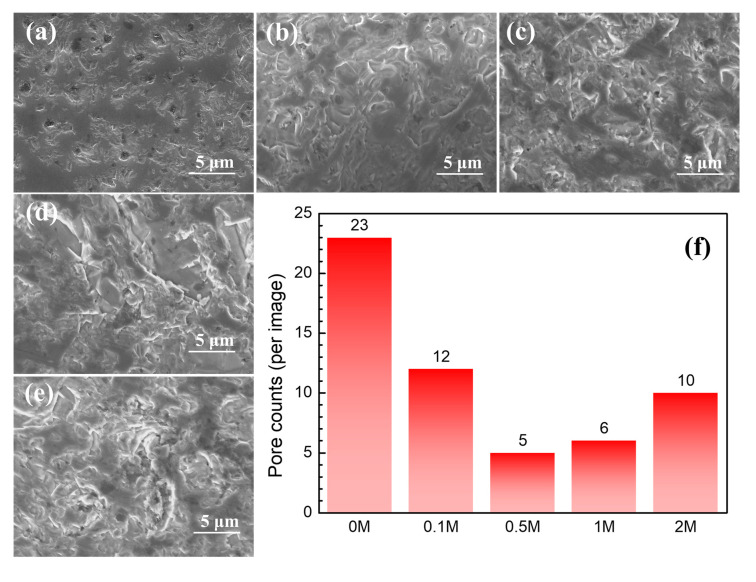
Effect of graphene doping on microstructure of SiC ceramics: (**a**) 0 M, (**b**) 0.1 M, (**c**) 0.5 M, (**d**) 1 M, (**e**) 2 M and (**f**) pore counts in different samples (randomly areas with the same scanning size).

It can be observed that the doping amount of graphene greatly influenced the porosity, grain boundary, and density of SiC ceramics from the microstructure morphology and the number of pores (randomly selected scanning areas with the same size on different samples) presented by SEM. In the 0 M samples without graphene (Figure 4a), grains were connected tightly, and the grain boundaries were fuzzy. The sections were relatively neat, mainly transgranular fractures, and many pores were observed. In the 0.1 M sample doped with a small amount of graphene (Figure 4b), a small number of grain boundaries can be observed, the porosity was less than that of the 0 M, and the ceramics were denser.

With the doping amount of graphene in the 0.5 M sample (Figure 4c) further increasing, the pores became smaller, the sample was denser, and the number of grain boundaries that could be observed increased. In the 1 M sample (Figure 4d), the number of grain boundaries observed further increased, and the number of pores also increased slightly in the uneven sections from the intercrystalline fracture surfaces. When the graphene amount increased to 2 wt.%, more grain boundaries and pores were observed in the 2 M sample (Figure 4e). The section was almost all intergranular fracture, with a grain size of 1–5 μm, which was consistent with the size of SiC powder particles.

### 3.5. EPMA Element Mapping

**Figure 5:**
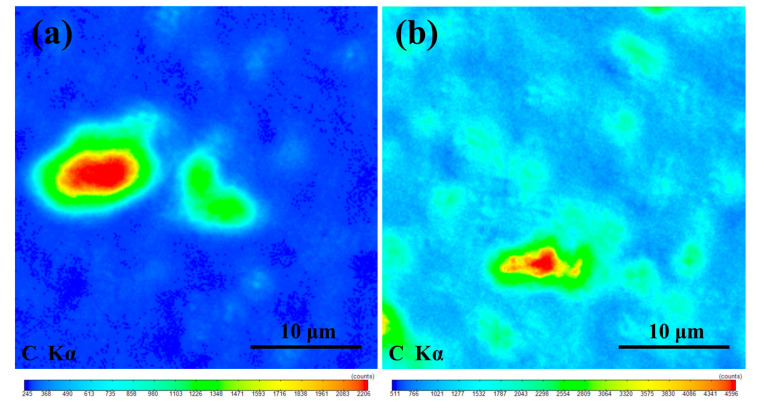
EPMA element mapping results of (**a**) 0.1 M and (**b**) 2 M for carbon.

Electron probe microanalysis (EPMA) was used to analyze the distribution of carbon elements in the micro regions of 0.1 M and 2 M samples. The area in the image was randomly selected, with a size of 45 μm × 45 μm. The signal color changed from blue to red, indicating the intensity of the carbon content, as shown in the scale bars in Figure 5a,b. The closer the signal color was to the red side, the higher the carbon content in the area. In the 0.1 M (Figure 5a), the carbon elements in the SiC grains were evenly distributed, the contrast signal was weak, and they were interconnected, forming a region like the “sea”. The carbon concentration area, the contrast signal was strong, forming an “island” region. The island was thought to have been formed after the graphene-filled pores in SiC ceramics.

In the 2 M (Figure 5b) with a large amount of graphene doped, in addition to the sea with uniform distribution of carbon elements and the island with concentrated distribution, many “shoals” with a dispersed distribution of carbon elements in multiple regions were also detected. These shoals connected the islands.

### 3.6. Raman Spectroscopies

**Figure 6:**
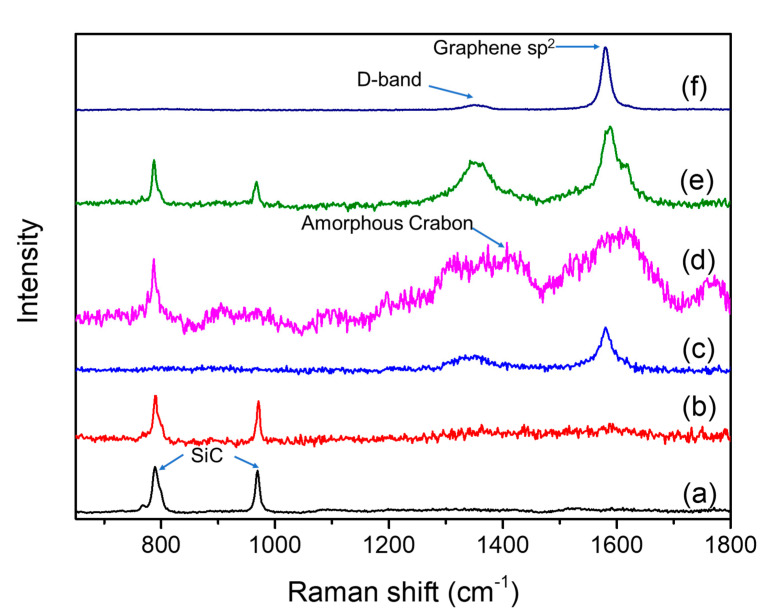
Raman spectroscopies of the samples: (**a**) in the grain of 0 M; (**b**) in the grains of 0.1 M; (**c**) intergranular of 0.1 M; (**d**) in the grain of 2 M; (**e**) intergranular of 2 M; and (**f**) freshly prepared graphene suspension.

The phase composition of the sea, shoals, and island revealed in the distribution of carbon elements needs to be defined. According to the test results of Raman spectroscopy of the micro-regions in SiC ceramics, two characteristic peaks of SiC about 785 and 965 cm^−1^ were detected on the fracture surface of 0 M (Figure 6a), and no Raman peaks of other materials were detected. On the surface of 0.1 M (Figure 6b), in addition to the characteristic peaks of SiC, the Raman peaks of graphene were also vaguely detected. Between the grains of 0.1 M (Figure 6c), the characteristic peaks of graphene about 1355 and 1580 cm^−1^ were directly detected as freshly prepared graphene suspension (Figure 6f). Therefore, it can be confirmed that, in 0.1 M, a certain amount of graphene filled the pores of SiC ceramics to lead to densification and showed island-like carbon element distribution characteristics in EPMA testing. 

The signature peaks of graphene with stronger signals were detected between the grains of 2 M (Figure 6e) and on the surface of the 2 M grains (Figure 6d). Moreover, the amorphous carbon peak indicated an increase in carbon content and no longer maintains the graphene state. The EPMA analysis showed that, with the increasing of graphene, the remaining graphene “spills” into the SiC ceramic grain boundary after filling the pores. During ceramic sintering, the graphene between the grain boundaries was extruded by the grains and spread out; they can be connected to each other to form a spatially connected network. When the ceramics broke, the graphene between the grain boundaries was exposed and detected.

### 3.7. Mechanism

According to the abovementioned experimental and characterization results, the doping amount of graphene affected the porosity and grain boundary of SiC ceramics, and further affected the density and flexural strength of SiC ceramics. Combined with various testing characterization, the mechanism was analyzed as follows. Porosity and grain boundary are the key factors that influence the density and strength of SiC ceramics [24,25,26]. Graphene has excellent mechanical properties, but its density is very low. During the sintering of SiC ceramics, the graphene did not melt into the SiC ceramic grains. On the contrary, it was squeezed into the pores between the grains during the ceramic’s densification. A small amount of graphene filled up the pores between the grains of SiC ceramics and improved the mechanical strength of the ceramic. Therefore, the flexural strength of 0.1 M was higher than that of 0 M. However, further increasing of the graphene caused the extra graphene to enter the SiC ceramic grains as a heterogeneous phase, blocking the mutual bonding of homogeneous SiC grains, which led to more grain boundaries and pores. When the SiC ceramic was subjected to external force, the crack was easily spread, along with the interface between the SiC and the graphene, and caused the SiC ceramics to fracture. Therefore, when graphene was excessive, the flexural strength of SiC ceramics decreased gradually as the doped amount of graphene increased. 

Meanwhile, the volume resistivity of SiC ceramics could be regulated by the doping amount of graphene, which resembles the interfacial resistance [27,28] or interfacial conduction model [29,30]. In 0.1 M, which doped very little graphene, the highly conductive graphene filled in the pores of the SiC ceramics and formed “nodes” (“islands” in EPMA), which could facilitate high-speed electron transfer. These conductive “nodes” were irregularly dispersed in ceramics. Although these “nodes” were isolated, they effectively reduced the barriers (pores), which interfere with electron transport. Moreover, these “nodes” resulted in the resistivity of 0.1 M being two orders of magnitude lower than that of 0 M. 

As the amount of graphene further increased, graphene entered into the grain boundary of SiC ceramics and became more and more widely distributed. It promoted the connection of some isolated “nodes” and gradually formed a continuous two-dimensional network with cluster structures (“shallow” in EPMA), namely the “high-speed channel” of local electron transmission. 

When the doping of graphene reached a certain amount, the electron transmission of the “high-speed channels” could be connected to each other by graphene in grain boundary, forming a three-dimensional conductive network inside the whole block of SiC ceramics, meaning that the electron exceeded the minimum of percolation [31,32,33] and led to a further reduction in the volume resistivity of SiC ceramics. Once the electron transport barrier is broken by graphene conductive channels, further increase of the doping amount of graphene would not lead to a significant decrease in the resistivity. Therefore, the resistivity reduction of 1 M and 2 M tended to be flat.

## 4. Conclusions

In this study, a low resistivity pressureless sintering of SiC was fabricated by doping graphene into SiC as conducting grain boundary phases. The volume resistivity of the composite decreased logarithmically, while the bulk density decreased gradually, with the increasing of graphene from 0 to 2 wt.%. When the amount of graphene was smaller than 1 wt.%, graphene filled into the pores in the pressureless sintered SiC. This phenomenon enhanced the mechanical strength and decreased the resistivity of the composite significantly. When the amount of graphene was larger than 1 wt.%, graphene formed a spatially connected network in the SiC ceramics. Although the resistivity was further reduced, the volume density bulk density and flexural strength of the composite were greatly reduced, which may not meet the requirements of friction pair materials for mechanical seals. To decrease the resistivity of SiC ceramics without losing its excellent mechanical properties for mechanical seals, the additive amount of graphene was optimized to 1 wt.%. The resulting volume resistivity of the composite was of 397 Ω·cm, the bulk density was 3.076 g/cm^3^, and the flexural strength was 364 MPa. It is possible to fabricate low-resistivity SiC ceramic as a useful friction pair material for mechanical seal in a special condition, without excessively losing its excellent mechanical properties by the introduction of partially connected graphene as conductive pathways into semiconducting ceramic.

## Figures and Tables

**Table 1:** Effects of graphene doping on SiC ceramics.

Samples Number	Doped Amount of Graphene (wt.%)	Bulk Density (g/cm^3^)	Flexural Strength (MPa)	Volume Resistivity (Ω·cm)
0 M	0.00	$3.132_{-0.003}^{+0.002}$	$390_{-1}^{+2}$	>10^6^
0.1 M	0.10	$3.108_{-0.002}^{+0.004}$	$410_{-2}^{+3}$	1.2 × 10^4^
0.5 M	0.50	$3.088_{-0.003}^{+0.003}$	$384_{-3}^{+2}$	1.2 × 10^3^
1 M	1.00	$3.076_{-0.002}^{+0.003}$	$364_{-2}^{+4}$	$397_{-5}^{+5}$
2 M	2.00	$3.039_{-0.003}^{+0.002}$	$339_{-1}^{+3}$	$192_{-5}^{+5}$
